# Photocatalytic and Antimicrobial Activities of Biosynthesized Silver Nanoparticles Using *Cytobacillus firmus*

**DOI:** 10.3390/life12091331

**Published:** 2022-08-28

**Authors:** Ebrahim Saied, Amr H. Hashem, Omar M. Ali, Samy Selim, Mohammed S. Almuhayawi, Mostafa A. Elbahnasawy

**Affiliations:** 1 Botany and Microbiology Department, Faculty of Science, Al-Azhar University, Nasr City, Cairo 11884, Egypt; 2 Department of Chemistry, Turabah University College, Turabah Branch, Taif University, P.O. Box 11099, Taif 21944, Saudi Arabia; 3 Department of Clinical Laboratory Sciences, College of Applied Medical Sciences, Jouf University, Sakaka 72341, Saudi Arabia; 4 Department of Medical Microbiology and Parasitology, Faculty of Medicine, King Abdulaziz University, Jeddah 21589, Saudi Arabia; 5 Yousef Abdulatif Jameel Scientific Chair of Prophetic Medicine Application, Faculty of Medicine, King Abdulaziz University, Jeddah 21589, Saudi Arabia

**Keywords:** industrial wastewater, silver nanoparticles (Ag-NPs), photocatalytic degradation, phytotoxicity, methylene blue (MB) dye

## Abstract

The toxicity of the ecosystem has increased recently as a result of the increased industrial wastewater loaded with organic contaminants, including methylene blue (MB), which exerts serious damage to the environment. Thus, the present work aims to green the synthesis of silver nanoparticles (Ag-NPs) and to evaluate their degradability of notorious MB dye, as well as their antimicrobial activities. Ag-NPs were synthesized by *Cytobacillus firmus* extract fully characterized by UV-vis, TEM, DLS, XRD, and FTIR. Ag-NPs showed good antibacterial and antifungal activities against *Escherichia coli* ATCC 25922, *Enterococcus feacalis* ATCC 29212, *Pseudomonas aeruginosa* ATCC 27853, *Staphylococcus aureus* ATCC 25923, and *Candida albicans* ATCC 90028. Moreover, Ag-NPs exhibited a high biodegradability level (98%) of MB dye after 8 h of co-incubation in the presence of sunlight. Additionally, the phytotoxicity of treated MB dye-contaminated water sample showed good germination of *Vicia faba* as compared with non-treated MB dye-contaminated solution. In conclusion, the herein biosynthesized Ag-NPs demonstrated its feasibility of the purification of contaminated water from microbes and methylene blue dye and the probability of reusing purified water for agricultural purposes.

## 1. Introduction

Water pollution has become one of the most serious social problems in recent decades as a result of the widespread use of dangerous chemicals such as organic dyes. Organic dyes are non-biodegradable and toxic chemicals for aquatic life and human health. Dye wastes have mutagenic and carcinogenic impacts on humans and considered significant polluters for the environment [1]. Therefore, there is an utmost need for removing these toxic dyes from water that will obviously lower water pollution levels. Indeed, there are numerous conventional biological and physic-chemical procedures for water treatment but not effective for removing dye residues [2]. That is due to the high stability and complicated structures of dyes. Traditional water treatment procedures include adsorption, ultrafiltration, coagulation, and reverse osmosis, which are frequently unable to decolorize or mineralize such toxic dyes. Therefore, new techniques for degrading these dangerous dyes are required [3]. Many hazardous dyes, such as methylene blue (MB), methyl orange (MO), acridine orange (AO), and rose bengal (RB) were degraded by using nanomaterials that exhibited good photocatalytic activity [4]. Removing dye impurities from wastewater by eco-friendly approaches, such as using bio-fabricated nanoparticles (NPs), has raised a lot of interest due to the fast oxidation, absence of formation of polycyclic compounds, and oxidation of pollutants [5]. Nanoparticles have been used for biomedical, agricultural, and environmental applications [6,7,8,9,10,11,12]. It is well established that the biological synthesis of NPs is cost-effective, biocompatible, non-toxic, and environmentally safe [13,14,15,16,17,18,19]. Unlike NPs made of synthetic chemical compounds, biogenic NPs have proved more stable at room temperature for extended periods of time, with no agglomeration [20]. Bacteria are a good choice for NP biosynthesis since they grow quicker than plants or fungi and their growing conditions can be manipulated and cost-effective [21,22]. Silver is a preferred metal for nanoparticle synthesis regarded to its antimicrobial and catalytic activities [23,24,25]. Recent studies have looked into the catalytic and dye degradation capability of Ag-NPs [26,27]. Dyes are used in a wide range of industries, including textiles, paper, leather, food, and cosmetics [28]. The most commonly used dye in cotton, wool, and silk is MB. Due to its toxicity and long-term persistence in the environment, it is critical to develop safe and effective methods for reducing the environmental impact of MB [29]. Wicaksono, Sahroni [30] investigated the formation of SnO_2_ nanoparticles by using Red Spinach (*Amaranthus tricolor* L.) extract and studying their photocatalytic activity for dye degradation. Moreover, Momin, Rahman [31] used silver nanoparticles from *Bacillus licheniformis* M09 for photocatalytic degradation of dyes and antibacterial activity studies. In general, the smaller the size of the nanoparticles, the greater the antimicrobial activities they could have due to the increased surface area [32]. This study aimed to develop a green method for bio-fabrication Ag-NPs by using *Cytobacillus firmus cell-free* extract and to evaluate their antimicrobial activity against pathogenic human microbes. Moreover, Ag-NPs were used to decolorize Methylene blue contaminated water. Finally, the phytotoxicity of treated water was evaluated for germination of *Vicia faba*.

## 2. Materials and Techniques

### 2.1. Isolation and Identification of Bacterial Strain

The strain was isolated at a depth of 5–10 cm in Egypt’s eastern desert (N: 29°58′49.06″, E: 32°7′3.39″). The bacterial strain was isolated using the serial dilution technique on Luria-Bertani (LB) agar plates and incubated at 37 °C for 24 h. The strain was identified by its morphological, physiological, and biochemical properties and 16S-rRNA sequencing, as previously described [33]. In summary, genomic DNA was extracted and primerss27F (5′-AGAGTTTGATCCTGGCTCAG-3′) and 1492R (5-GGTTACCTTGTTACGACTT-3′) were used to amplify for 16S-rDNA. PCR products were purified by pure link quick gel extraction kit and sequenced by ABI 3730xl DNA sequencer (GATC Biotech, Germany). Sequence consensus was compared with NCBI Gene Bank data base using the NCBI BLAST program. Phylogenetic studies were performed using MEGA 7.0 software with 1000 bootstrap repeats using the neighbor-joining approach based on 1000 replicates.

### 2.2. Ag-NPs Biosynthesis and Characterization

Ag-NPs were produced as previously reported [20]. The bacterial strain was isolated onto LB agar plates (1% tryptone, 0.5% yeast extract, 1% NaCl, 1.5% agar, and pH 7) by serial dilution plate technique at 37 °C. Then, it was sub-cultured into LB broth and incubated in an Orbital Shaker (37 °C and agitation at180 rpm). The microbial biomass was collected after 24 h and centrifuged at 20,000 rpm for 5 min. The cells were discarded and the supernatant (Cell-free extract “CFE”) was collected and mixed with a silver nitrate (AgNO_3_) solution (1 mM). Then, the mixture was incubated in a shaking incubator (200 rpm, 37 °C) for 24 h in dark conditions, to avoid oxidation of AgNO_3_, for the biosynthesis of AgNPs. As a result, the color of the solution turned from yellow to brown, which is a primary indicator for the formation of AgNPs.2.3. The effect of some parameters is conditioned on the biosynthesized Ag-NPs.

Different parameters, including silver nitrate (AgNO_3_) concentrations (1, 2, 3, and 4 mM), incubation time (6, 12, 24, 36, 48, and 72 h), and pH (6, 7, 8, 9, and 10), were chosen to optimize the best high yield for Ag-NPs. The experiment was carried out in three replicates and the results were monitored using the UV-Vis. The experiment was designed by examining four different concentrations of silver nitrate (AgNO_3_) (1 mM, 2 mM, 3 mM, and 4 mM) solutions to find the optimal concentration substrate that can be converted into Ag-NPs. Incubation times of 6, 12, 24, 36, 48, and 72 h were chosen to select the best time to give high yield productivity of Ag-NPs.

### 2.3. Characterization of Optimized Ag-NPs

Ag-NPs were characterized as described previously [20]. The visual monitoring of Ag-NPs was done by comparing the brown color reaction to the controls, and the results were validated using a UV-visible spectrophotometer (scanning spectra range from 300 to 700 nm, Shimadzu UV-1700, Kyoto 604-8511, Japan). At a voltage of 200 kV, transmission electron microscopy (JEOL 1010 TEM, Japan) was employed to study the morphology of Ag-NPs. Dynamic light scattering (DLS) (The Nicomp ZLS Z 3000, Santa Barbara, CA 92154, USA) was used to estimate the average particle size and size distribution of Ag-NPs in colloidal solutions, with observations ranging from 0.1 nm to 10,000 nm. X-ray diffraction (XRD) (Shimadzu LabX XRD-6000) with a Cu-Kα X-ray source (λ = 1.5418 Å) analysis was used to evaluate the crystallite nature of Ag-NPs. The identification of functional groups that might be responsible for the reduction of silver ions to Ag-NPs was done using Fourier Transform Infrared (FTIR) Spectroscopy (Perkin-Elmer FTIR-1600, Mundelein, IL, USA). An aliquot of 300 μL of Ag-NPs which was mixed with potassium bromide (10 mg) was oven-dried. For purification the Ag-NPs colloids were high centrifuged, pellets were washed three times with deionized distilled water, filtered through a millipore filter to remove any residue of supernatant, dried at 40 °C (18–24 h), afterward collected and stored for characterization.

### 2.4. Antimicrobial Activities of Ag-NPs

Biosynthesized Ag-NPs were studied against five microbial strains, including *E. coli* aATCC 25922, *S. aaureus* ATCC 25923, *P. aeruginosa* ATCC 27853, *E. feacalis* ATCC 29212, and *C. albicans* ATCC 90028. Agar well diffusion method was performed according to protocol M51-A2 of the Clinical Laboratory Standard Institute [34] with minor modifications. Bacterial and unicellular fungi suspensions (1.5 × 10^6^ CFU mL^−1^) were prepared and seeded into Muller Hinton agar medium separately. AgNPs, standard antibiotic (Amoxicillin/clavulanate), and standard antifungal (Nystatin) at a concentration of 500 µg/mL were added in an agar well (7 mm) and incubated at 37 °C for 24 h for bacteria, and at 30 °C for 48 h for *C. albicans*. At the end of the incubation time, inhibition zone diameters were measured. Different concentrations (7.81 to 500 µg mL^−1^) of AgNPs, Amoxicillin/clavulanate were evaluated, and then tested independently for MIC against a selection of bacterial and fungal species [35].

### 2.5. Photocatalytic Degradation of Methyleneeblue Dye Solution by Ag-NPs

The degradation of MB dye was monitored at different concentrations of Ag-NPs (0.25, 0.5, 0.75, and 1.0 mg mL^−1^) and different contact times (2, 4, 6, 8, 10, and 12 h) under sunlight and dark conditions to investigate the photocatalytic effectiveness of biosynthesized Ag-NPs. The photocatalytic experiment was carried out in triplicates using 100 mL of MB dye solution at a concentration of 100 ppm, mixed with different Ag-NP concentrations, and incubated at room temperature under shaking conditions. Secondly, the same reactions were performed under dark conditions for comparison. The following criteria were used to evaluate the decolorization efficacy: 1.0 mL of each treatment was taken and centrifuged for 5.0 min at 5000 rpm, then the optical density (O.D.) of MB dye solution (630 nm) was measured by the spectrophotometer (721 spectrophotometers, M-ETCAL). The following equation: D = [Dye (i) − dye (I)/dye (i)] × 100, was used to measure the decolorization percentage (%) of MB dye [36], where D (%) is the decolorization percentage, dye (i) is initial absorbance, and Dye (I) is final absorbance.

### 2.6. Phytotoxicity Test

The phytotoxicity of Ag-NPs-treated MB dye solution was investigated using broad bean (*Vicia faba*) seeds. Healthy seeds were purchased from the local market and subjected to surface sterilization by soaking in 2.5% sodium hypochlorite for 5 min, then 70% (*v*/*v*) ethanol for 1 min, and finally washed with sterile distilled water 5 times. The following treatments were carried out: tap water as a control (i), MB dye solution before any treatment (ii), and MB dye solution after treatment with Ag-NPs (1.0 mg/mL, 8 h, and sunlight). Before the experiment, the chosen seeds were germinated in tap water until a 0.5 cm seed radical developed to indicate seed health, then transferred to a petri dish (25 cm) containing filter papers, moistened with treatments, and incubated at 30 °C. The growth parameters including shoot length and root length were calculated after 12 days of incubation. The experiment was carried out in triplicates.

### 2.7. Statistical Analysis

All results are presented as the means of three independent replicates. Data were statistically analyzed by SPSS software version 17. The mean difference between the treatments was analyzed by *t*-test or (ANOVA) and posteriorly by Tukey HSD test at *p* < 0.05.

## 3. Results and Discussion

### 3.1. Isolation and Identification of the Bacterial Isolate

**Figure 1:**
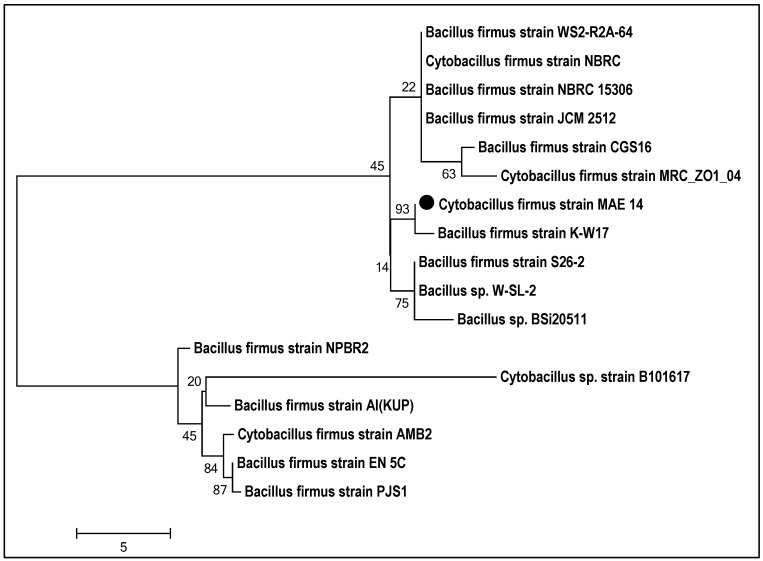
Identification of *Cytobacillus firmus* strain MAE14 by phylogenetic analysis. Neighbor-joining tree obtained by distance matrix analysis of 16S rRNA gene sequences, showing the position of *Cytobacillus firmus* strain MAE14 among phylogenetic neighbors of *Bacillus* spp. published in GenBank. Bootstrap values from 1000 replications are indicated at the branches.

The current study adopts an eco-friendly biological approach for the biosynthesis of (Ag-NPs). Identification of bacterial isolates was carried out by morphological and biochemical characteristic analysis, and then confirmed by molecular identification (16s rDNA). The bacterial strain MAE14 was morphologically characterized as Gram-positive, endospore formers, motile rods and arranged singly, in pairs, or in short chains. The biochemical assays of MAE14 were carried out by the bioMérieux VITAK2 system and exhibited the identical characteristics of *Cytobacillus firmus* regarding Bergey’s Manual of Systematic Bacteriology as seen in supplementary Table S1. For further confirmation, the molecular identification was performed by sequencing of the 16s rDNA fragment of MAE 14. Constructed phylogenetic tree of MAE14 exhibited clustering relationship among MAE14 and *Cytobacillus firmus* strains as shown in Figure 1. Hence, the bacterial isolate MAE14 was identified as *Cytobacillus firmus* strain MAE14 (Figure 1) and the sequence was deposited in Gene Bank under accession number MW509089.1.

### 3.2. Green Biosynthesis of Ag-NPs

**Figure 2:**
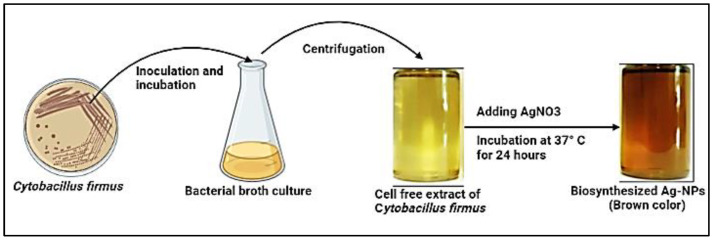
Biosynthesis method of Ag-NPs using CFE of *Cytobacillus firmus*.

**Figure 3:**
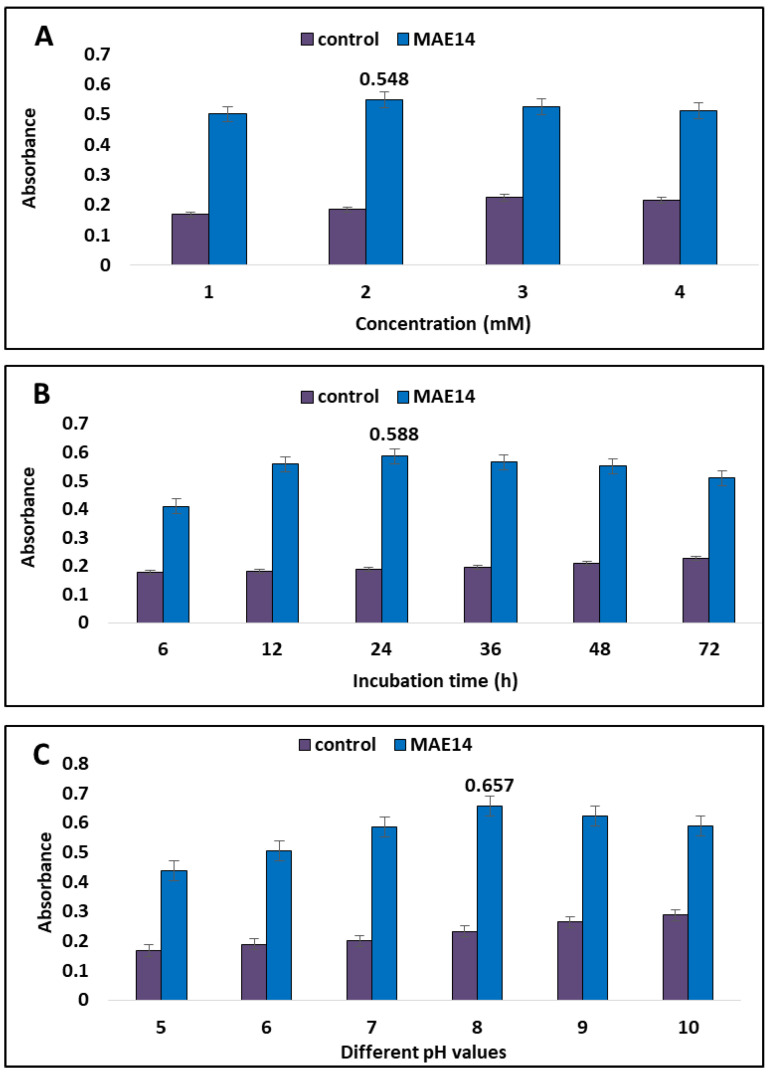
Optimizing factors for synthesis of Ag-NPs using *Cytobacillus firmus* strain MAE14 (**A**) denotes the different AgNO_3_ concentrations; (**B**) denotes the contact time between biomass filtrate and optimum AgNO_3_ concentration; (**C**) illustrates the effect of pH values.

In the current study, cell-free extract of *Cytobacillus firmus* strain MAE14 was incubated with AgNO_3_ as the precursor of Ag-NPs. Thus, Ag^+^ were electrostatically attracted toward the OH groups of bioactive metabolites in cell-free extract and in turn, Ag^+^ ions were reduced to Ag^0^ leading to the formation of Ag-NPs. Visual observation of the final end product (Ag-NPs) was brown and the control showed no change in color (Figure 2). The optimal physicochemical parameters for high-yielding silver nanoparticles were studied. Optimization parameters include the concentration of AgNO_3_, reaction incubation time, and pH values. The results revealed that the maximum high yield of silver nanoparticles occurred at 2 mM of silver nitrate and pH 8 after 24 h of incubation time (Figure 3). Furthermore, exposure to an excess of reductase enzymes that may be accessible at lower AgNO_3_ concentrations may be the optimal silver nitrate concentration for the synthesis of silver nanoparticles, but higher production may not have happened owing to a limitation of substrate molecules. Furthermore, the presence of substrate in the media may trigger the enzyme to be released. Moreover, high silver salt concentrations can lead to aggregated nanoparticles. Alsamhary [37] showed that aqueous silver ions (Ag^+^) were transformed to Ag-NPs after being introduced to the cell-free supernatant of *Bacillus subtilis* for 18 h at a concentration of 1 mM silver nitrate. Similarly, Esmaile, Koohestani [38] observed that the synthesis of Ag-NPs occurred at pH 8 by using *Ziziphora clinopodioides*. Alternately, the best conditions for the synthesis of Ag-NPs, were 10 g fungal biomass, a reaction temperature of 28 °C for a 72 h incubation period, and no shaking according to Elamawi, Al-Harbi [39]. Ghiuță, Cristea [40] produced Ag-NPs after 48 h of incubation between biomass and its precursor. Rajkumar, Ezhumalai [41] reported that the optimum pH was 12 for synthesis of Ag-NPs by using *Chlorella vulgaris* at a concentration of 3 mM of AgNO_3_ after 24 h. Wypij, Jędrzejewski [42] succeeded to synthesize Ag-NPs by actinobacterial strain SF23 in dark conditions for 3 days at 25 °C. Giri, Jena [43] produced Ag-NPs after 48 h of incubation between *Eugenia roxburghii* leaf extract and 0.1 mM AgNO_3_.

### 3.3. Characterization of Biosynthesized Ag-NPs

#### 3.3.1. UV-Visible Spectroscopy Analysis

**Figure 4:**
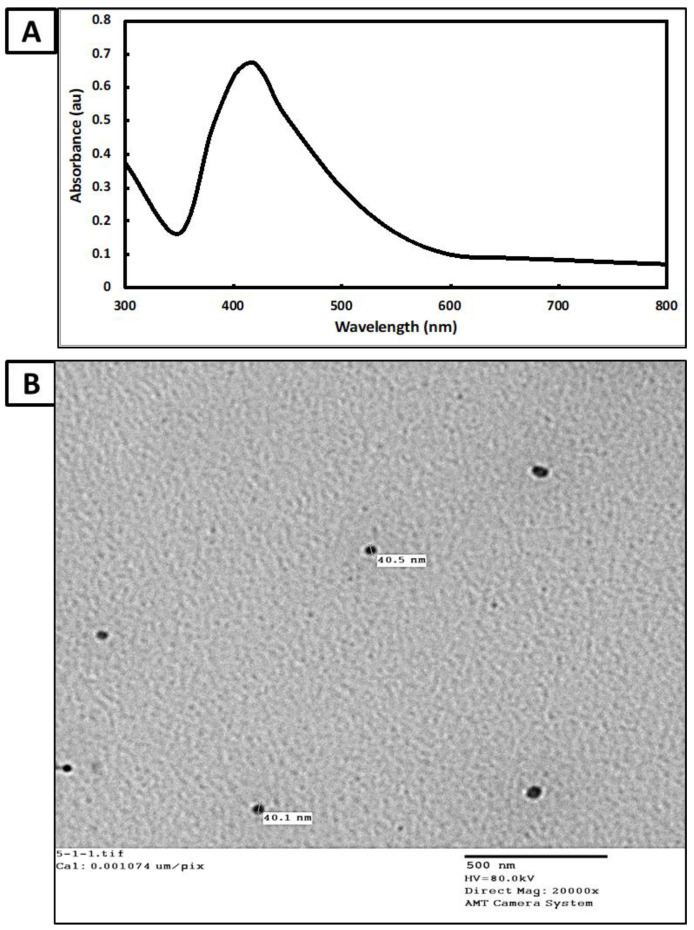
Characterization of biosynthesized Ag-NPs, (**A**) UV-vis spectra at wavelength 300–800 nm; (**B**) TEM image for Ag-NPs showing spherical shapes.

The strongest surface plasmon resonance (SPR) was detected using UV-visible spectroscopy to monitor the color intensity as an indication of Ag-NPs productivity. The highest SPR for Ag-NPs generated by *Cytobacillus firmus* MAE14 was 415 nm, according to data analysis (Figure 4A). The shape and size of Ag-NPs are generally linked to their given SPR state, as described before [44]. Herein, the SPR absorption band for *Cytobacillus firmus* MAE14 mediated Ag-NPs was detected at 415 nm, this is due to the mutual vibration of Ag-NPs free electrons in resonance with the light wave [45]. SPR values below or higher than 400 nm indicate smaller or bigger nanoparticles, respectively [46]. In line with our data, Alsamhary [37] presented a broad peak (400–470 nm) in the UV-visible spectrum for Ag-NPs produced by *Bacillus subtilis*.

#### 3.3.2. Transmission Electron Microscopy (TEM)

TEM analysis was applied to characterize the shape and size of produced Ag-NPs. The particles were almost spherical in form and monodispersely scattered without much aggregation, according to TEM micrographs (Figure 4B). The presence of biomolecules and other metabolites in biomass filtrate, which are utilized in the bio-reduction and bio-capping of produced nanoparticles, might be the origin of the variation in nano-size and nano-shape [47]. The TEM image exhibits well-dispersed Ag-NPs without any aggregation and the nanoparticle sizes ranged from 30 to 60 nm. The obtained data are consistent with Saeed, Iqbal [48] who successfully synthesized well-dispersed, spherical Ag-NPs, with a size range of 5–50 nm by using *Brevundimonas diminuta.* Thus, under lighting, AgNO_3_ can be reduced in the reaction mixture due to electrons that jump between energy levels forming Ag-NPs. Hamouda, Hussein [49], who employed *Oscillatoria Willei* NTDM01 extract for producing spherical Ag-NPs with sizes ranging from 100–200 nm.

#### 3.3.3. Dynamic Light Scattering (DLS) Analysis

**Figure 5:**
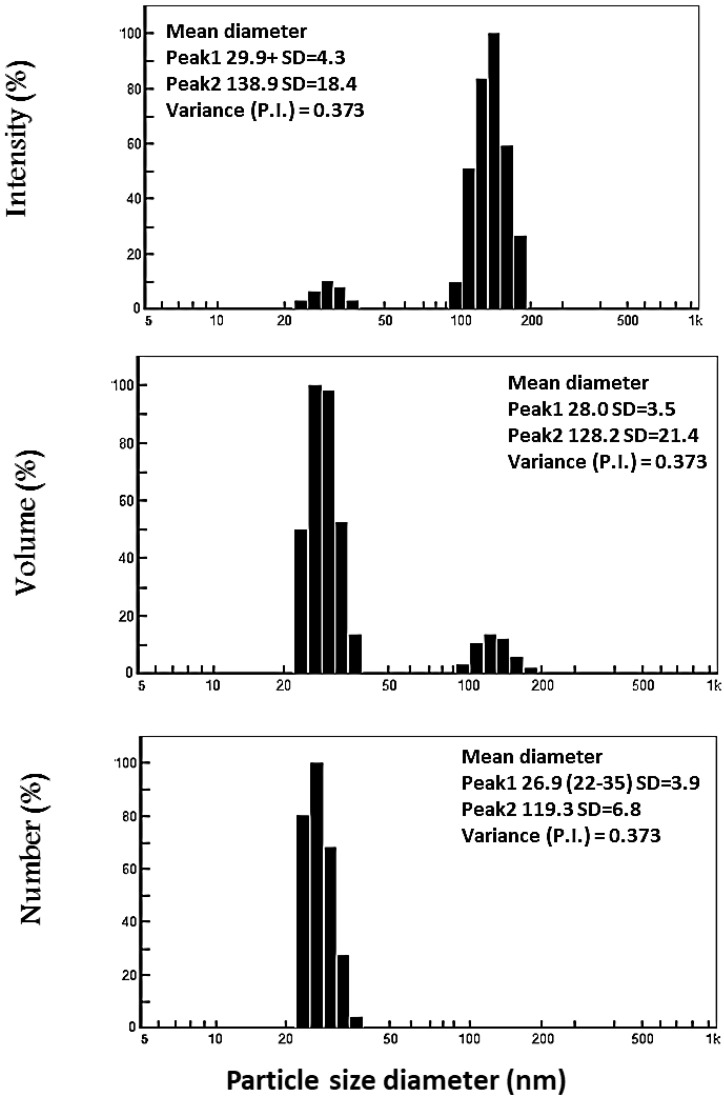
Size distribution of Ag-NPs by DLS.

DLS analysis was used to evaluate the size and particle distributions of Ag-NPs. As shown in Figure 5, the average hydrodynamic particle diameters for Ag-NPs were 55.8 nm for a volume. The average size produced by the DLS was larger than those generated by TEM analysis. This could be attributed to the electrical double layer formed on charged Ag-NPs and/or coating metabolites on the surface of the nanoparticles used for capping and stabilizing the Ag-NPs [50]. According to Mohmed, Saad [51] the average diameter of the Ag-NPs generated by *Aspergillus* sp. was determined to be 76.45 nm (97.4%). By evaluating the polydispersity value, the DLS analysis offers additional information regarding the homogeneity of particles in the colloidal solution (PDI). As previously reported, homogeneity was raised or decreased depending on the PDI value, with increased homogeneity if the PDI value was less than 0.4 and decreased homogeneity if the PDI value was greater than 0.4 [52]. When the PDI value exceeds 1.0, the solution becomes more diverse. In this study, the PDI value of biosynthesized Ag-NPs was 0.3, confirming that the Ag-NP colloidal solution was homogenous.

#### 3.3.4. X-ray Diffraction (XRD)

**Figure 6:**
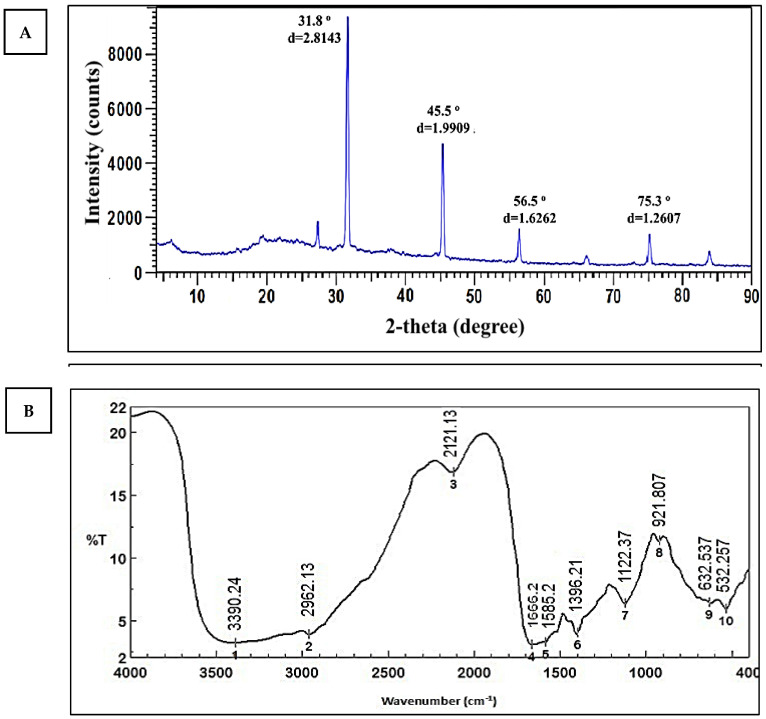
XRD spectrum of Ag-NPs (**A**), and FT-IR spectra of final product Ag-NPs (**B**).

The XRD pattern revealed four strong peaks at 2Ɵ of 31.8°, 45.5°, 56.5°, and 75.3°, corresponding to the planes (111), (200), (220), and (311). (Figure 6A). The preparation of Ag-NPs from *Cytobacillus firmus* MAE14 was demonstrated, and Ag-NPs were crystal and structure face-centered cubic in nature. The presence of some little peaks indicates that the sample has some impurities [53]. The Debye–Scherrer equation may be used to calculate the crystal size of Ag-NPs using the XRD pattern. The average crystal size of Ag-NPs was 38.3 nm, according to the data. This result is in agreement with Mohmed, Saad [51] who found that Ag-NPs biosynthesized using *Aspergillus* sp. and had diffraction planes (111, 200, 220, and 311). The result is proof that proteins play a key role in the fabrication of silver nanoparticles and act as capping and stabilizer agents in the biosynthesis of AgNPs. A wide variety of materials, including inorganic catalysts, superconductors, biomolecules, glasses, polymers, and more, may have their structural characteristics analyzed using XRD. When chemical substances are used as reducing agents, Ag^+^ is reduced to metallic silver (Ag^0^), which is followed by agglomeration into oligomeric clusters. Colloidal silver particles made of metal ultimately develop from these clusters. During the preparation process for metal nanoparticles, it’s essential to utilize protective agents to stabilize dispersive NPs, protect NPs that can bond to or absorb onto nanoparticle surfaces, and prevent NP agglomeration. The presence of Surfactants having functions for interacting with particle surfaces, such as thiols, amines, acids, and alcohols, can help to stabilize particle development and protect particles from sedimentation, agglomeration, or losing their surface features [54]. So that the shape, size, and monodispersity of the nanoparticles may be controlled by utilizing bacterial protein or plant extracts as reducing agents [55]. These results are in agreement with other reports used for biosynthesis of Ag-NPs [53]. El-Gamal, Salem [56], showed that intense peaks corresponding to (111), (200), (220), and (311), which exhibit the formation of Ag-NPs synthesized by *Streptomyces* sp. Ml-3, were crystal and structured face-centered cubic in nature. The consistency of these peaks was done by the High Score 2 software by comparing them with a database of pure crystalline silver. These results corroborate those reported in other studies [57].

#### 3.3.5. Fourier Transform Infrared (FT-IR) Spectroscopy

FT-IR measurements were carried out to acquire information about chemical groups present around Ag-NPs about their stabilization and understand the transformation of functional groups due to the reduction process. Data represented in (Figure 6B) revealed intense absorption peaks at 3390.24, 2962.13, 2121.31, 1666.2, 1585.2, 1396.21, 1122.37, 921.807, 632.537 and 532.257 cm^−1^. The broad peak at 3390.24 cm^−1^ corresponds to the O–H stretching group of phenols and alcohols. The peaks at 2962.13 and 2121.31 cm^−1^ may be corresponding to C–H vibrational stretching of aliphatic methyl (CH_3_) and methylene (CH_2_) groups [25]. The band at 1666.2 cm^−1^ may be corresponding to the binding of the amide I band of protein with the N–H stretching [58]. Two bands observed at 1396.21 and 1122.37 cm^−1^ can be attributed to C–N stretching vibrations of the aliphatic and aromatic amines [59]. Two bands at 921.807 and 632.537 cm^−1^ indicated the amide IV (OCN) stretch bending for proteins. The band at 532 cm^−1^ may be ascribed to alkene (=C–H bending) [60]. This amide I band indicates that proteins may be present in cell-free biomass filtrate of *Cytobacillus firmus* MAE14 and can bind to Ag^+^ ions across carboxylate ions or free amine groups to help in the biosynthesis of Ag-NPs in a reducing process. Other data have suggested that proteins could act as capping and/or stability agents in the biosynthesis of Ag-NPs [42,61]. El-Gamal, Salem [56], showed that FTIR spectra of endophytic *Streptomyces* sp Ml-3 mediated the synthesis of various silver nanoparticles were used to identify biomolecules involved in the reduction of Ag^+^ ions and capping of biosynthesized AgNPs. According to FTIR spectrum analysis, Elbahnasawy, Shehabeldine [62] found that the cell-free extract of *Rothia endophytica* MAE 11 might serve as reducing and stabilizing agents during the synthesis of Ag-NPs.

#### 3.3.6. Antimicrobial Activity

**Figure 7:**
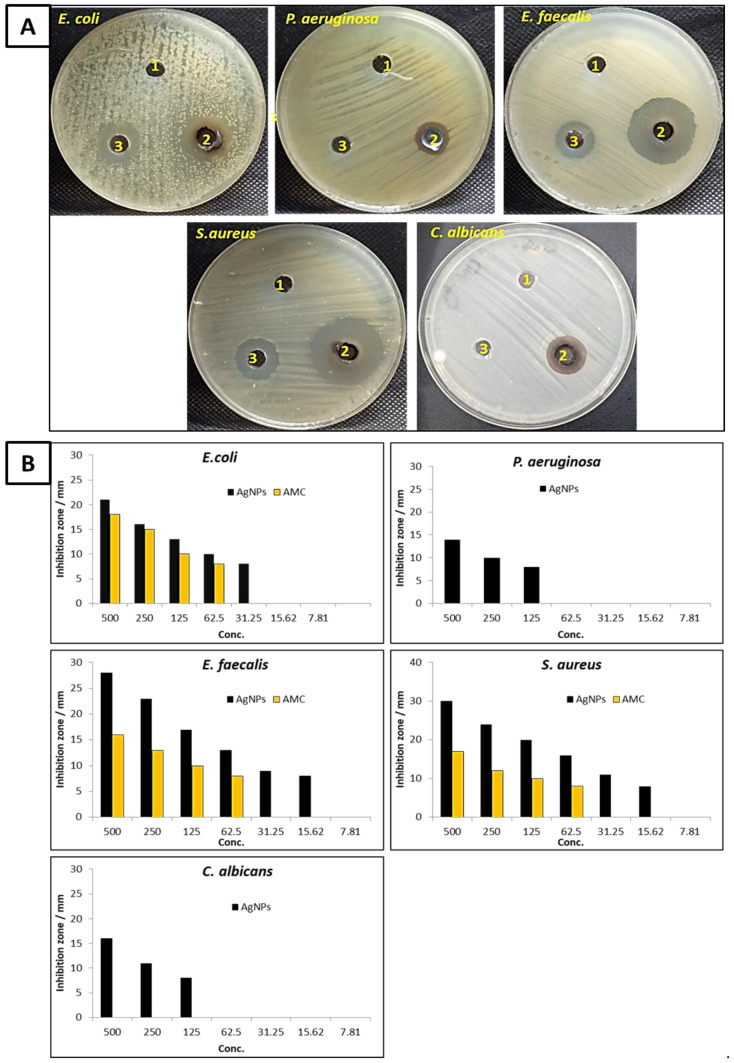
(**A**) antimicrobial activity of AgNO_3_ (1), Ag-NPs (2), and AMC/NS (3) at a concentration of 500 µg mL^−1^ against *E. coli*, *P. aeruginosa*, *E. faecalis*, *S. aureus*, and *C. albicans*; (**B**) effect of different concentrations of Ag-NPs and AMC/NS to detect MIC.

Figure 7 shows the antimicrobial activity of biosynthesized Ag-NPs against *E. coli*, *P. aeruginosa*, *E. faecalis*, *S. aureus*, and *C. albicans*. According to the results, the biosynthesized Ag-NPs containing endophytic *Cytobacillus firmus* extract had potential antibacterial activity against Gram-positive, Gram-negative, and unicellular fungi. Figure 7A,B illustrates the inhibition zone of biosynthesized Ag-NPs at a concentration 500 µg mL^−1^ against *E. coli*, *P. aeruginosa*, *E. faecalis*, *S. aureus* were 21, 14, 28, and 30 mm, respectively. These findings indicate that biosynthesized Ag-NPs have an effect toward Gram-positive than Gram-negative bacteria. Likewise, biosynthesized Ag-NPs exhibited antifungal activity against *C. albicans* where the inhibition zone was 16 mm. Furthermore, the MIC_s_ of Ag-NPs against *E. faecalis*, *S. aureus*, *E. coli*, *P. aeruginosa*, and *C. albicans* were determined as shown in Figure 7B. Both *E. faecalis* and *S. aureus* were the most sensitive microorganisms among the tested microbial strains, where MIC Ag-NPs for both was 15.62 µg mL^−1^. On the other hand, *E. coli*, *P. aeruginosa*, and *C. albicans* were less sensitive than *E. faecalis* and *S. aureus.* Meanwhile, the MIC_s_ of Ag-NPs toward *E. coli*, *P. aeruginosa*, and *C. albicans* were 31.25, 125, and 125 µg mL^−1^, respectively. Previous studies reported that the biosynthesized Ag-NPs have antimicrobial activity against pathogenic microbes [45,62]. Elbahnasawy, Shehabeldine [62] reported that the biosynthesized Ag-NPs using endophytic *Rothia endophytica* has activity against *C. albicans* where MIC and MBC were 62.5 and 125 µg mL^−1^. Similar to the result, Sudarsan, Kumar Shankar [45] used endophytic *Cytobacillus firmus* to biosynthesize Ag-NPs and discovered that Ag-NPs exhibited antibacterial efficacy against *E. coli* and *S. aureus*. Endophytic *Enterobacter hormaechei* was used for the biosynthesis of Ag-NPs that have antimicrobial activity toward *B. cereus* and *C. albicans* [63]. Moreover, biosynthesized Ag-NPs by endophytic *B. cereus* exhibited antibacterial activity against most common human pathogenic bacteria such as *E. coli*, *P. aeruginosa*, *S. aureus*, *Klebsiella pneumoniae*, and *Salmonella typhi*. The mechanism of action of biosynthesized Ag-NPs was carried out by silver ions that can be attached to the cell wall and cytoplasmic membrane, increasing the affinity to sulfur proteins and electrostatic attraction. The attached ions might increase the permeability of the cytoplasmic membrane, causing the bacterial envelope to be disrupted [64]. Increasing lipid peroxidation leads to cell membrane damage where leakage of cellular reducing sugars and total proteins occurs [62]. Silver ions can also interact with the DNA’s sulfur and phosphorus, causing issues with DNA replication and cell reproduction. Furthermore, silver ions can prevent protein production by denaturing ribosomes in the cytoplasm [65].

### 3.4. Photocatalytic Degradation of Methylene Blue Dye Decolorization by Ag-NPs

**Figure 8:**
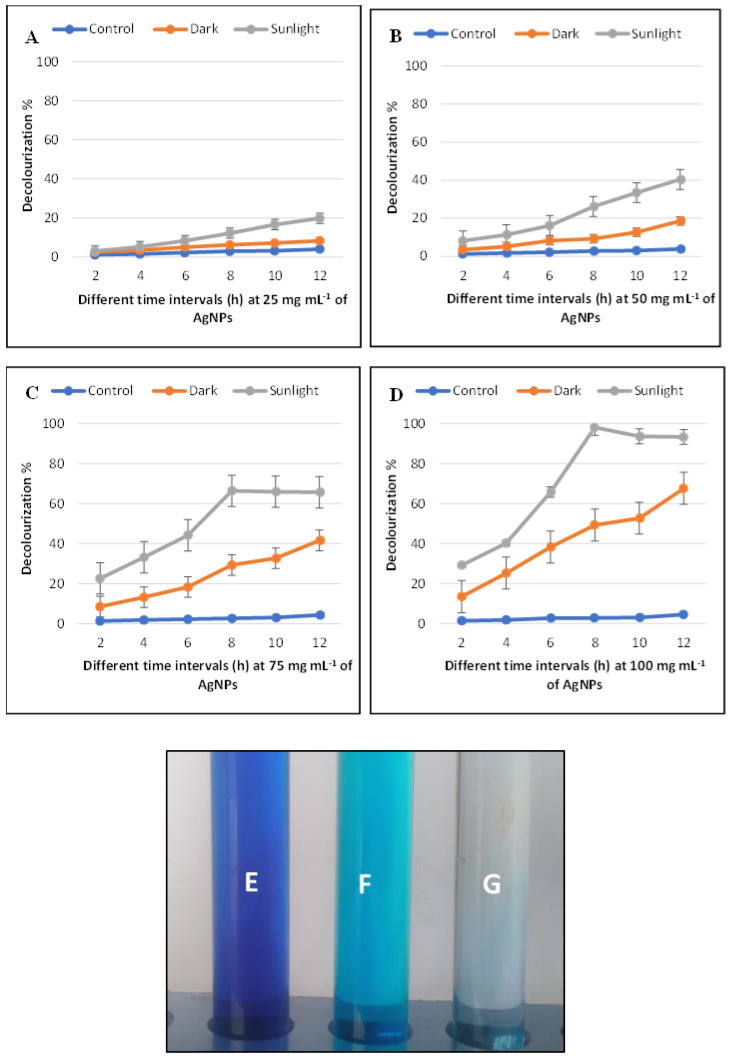
Methylene blue dye decolorization percentages at different Ag-NP concentrations 25 (**A**), 50 (**B**), 75 (**C**), and 100 (**D**) mg mL^−1^ at different contact times, and different stimulators ambient (Sunlight and dark condition). Dye decolorization under sunlight and dark condition, where (**E**), dye control; (**F**), dye under dark conditions and (**G**); dye under sunlight conditions.

The potential decolorization effects of Ag-NPs for methylene blue dye were examined at different concentrations of Ag-NPs (0.25, 0.5, 0.75, and 1.0 mg mL^−1^), different contact times (2, 4, 6, 8, 10, and 12 h), and under sunlight and dark conditions. Data exhibited that the catalytic activity of Ag-NPs was dose- and time-dependent. Remarkably, the biodegradation activity of Ag-NPs was much more stimulated in sunlight than that in dark conditions (Figure 8A–D). At concentration of 0.25 mg mL^−1^ for 12 h, decolorization (%) reached up to 19.8 ± 0.02% (sunlight) and 8.3 ± 0.01% (dark). In contrast, at 0.5 mg mL^−1^ of Ag-NPs, the decolorization percentages under sunlight stimulation were from 8% ± 0.02% after 2 h and reached up to 40 ± 0.03% after 12 h. The highest decolorization was achieved at 1.0 mg mL^−1^ of Ag-NPs concentration in the presence of sunlight with percentages of 98 ± 0.065% after 8 h, whereas in the dark ambiance, the same NPs concentrations were 67.6 ± 0.002% after 12 h. According to these data, 1.0 mg mL^−1^ of Ag-NPs after 8 h of contact time was therefore selected as the optimal condition. Fouda, Hassan [66], investigated the efficacy of biosynthesized MgO-NPs in textile wastewater degradation should be achieved in the presence of light as stimulators. Pandiyan, Dharmaraj [67], demonstrated that the biodegradation of dye was done under visible conditions by using hydrothermally produced bimetallic Ag-Sn hybrid nanocomposite. The effectiveness of biosynthesized MgO-NPs in methylene blue dye degradation should be accomplished in the presence of light [68]. The highest dye decolorization is attributed to the increase of Ag-NPs concentration that is because of the increase in the adsorption sites on the NPs surface [57]. The time required for degradation of either pure dye is varied than the complex solution that has more types of dye [69]. According to Khan, Khan [70], the metal oxides of NPs can induce charge separation by absorbing light and generating holes that decrease or oxidize organic compounds such as organic dyes. The photocatalytic activities in this study are caused by the activation of biosynthesized Ag-NPs by sunlight stimulators, as opposed to the dark condition (Figure 8E–G).

### 3.5. Phytotoxicity of Methylene Blue Dye Solution

**Figure 9:**
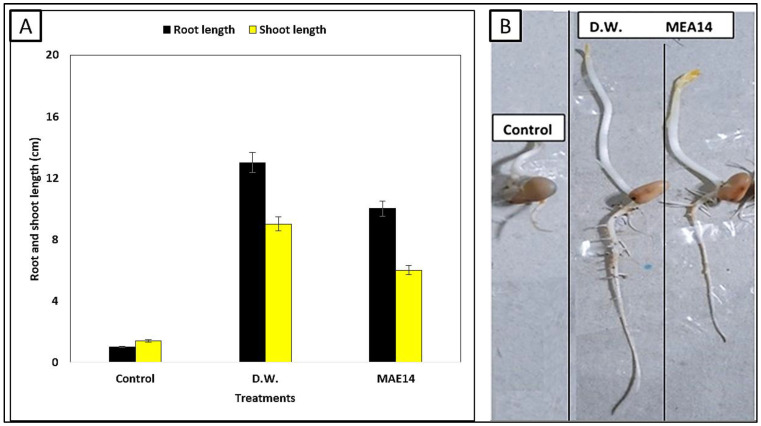
(**A**) Effect of MB dye before and after Ag-NPs treatment produced by *Cytobacillus firmus* MAE14 on root and shoot length of *V. faba* L. (**B**) Seed germination of broad bean irrigated by MB dye before and after Ag-NPs treatment. Control, methylene blue dye without any treatment; D.w, distilled water, and MAE14, treated by *Cytobacillus firmus* MAE14.

Phytotoxicity tests were conducted to assess the environmental effects of degrading solution discharge, as well as the possible use of the pre-treated aqueous solution in irrigation fields. Seed germination is the most common, simplest, sensitive, short-term, and cost-effective method used to evaluate the toxicity of dyes [35]. The toxicity of MB dye is correlated with its physicochemical characterization. MB dye exerts negative impacts on crops [71]. The remaining salts, organic residues, and other contaminants may be to blame for the decrease in root length irrigated by MB treated by NPs as compared to the tap water (control). To evaluate the effectiveness at the gene level, more investigation is needed for the phytotoxicity and genotoxicity studies [66]. In this work, we evaluated the potentiality of Ag-NPs under optimal conditions (1.0 mg mL^−1^/8 h/sunlight) in MB dye treatment to MB dye without any treatment using an important crop, broad bean (*Vicia faba*). The toxicity of the methylene blue dye is greatly reduced as a result of the Ag-NPs treatment, according to (Figure 9A,B). In comparison to untreated effluents, the root length of the broad bean resulting from the treatment with distilled water was 13 cm. In contrast, the root length of the broad bean did not develop around 1 cm owing to MB dye only. Furthermore, untreated MB dye is very harmful to broad bean shoot length, and there was no growth response of about 1.4 cm. Interestingly, when comparing the shoot and root length of the broad bean treated with MB dye treated by Ag-NPs to the shoot and root length moistened with distilled water, there was a significant difference. The residual salts, organic residues, and other contaminants might be to responsible for the reduced root length moistened by effluents treated by Ag-NPs as compared to distilled water [66]. In another study, the germination index reaches its highest value (75%) after 48 h, according to Venkatesh and Sakthivel [72], where the toxicity of the treated solutions is minimal. Finally, the findings suggested that seed germination might help in the reuse of treated water. The seed germination is an indication of the improvement in the quality of the treated water, and therefore it can be used again in the irrigation of ornamental plants or re-used again in textile factories.

## 4. Conclusions

Herein, silver nanoparticles (Ag-NPs) were produced by cell-free extract of Cytobacillus firmus strain MAE14, then they were characterized by UV–Vis spectroscopy, TEM, FTIR, XRD, and DLS analysis. The formation of Ag-NPs was dependent on the reaction time, pH, and precursor (AgNO3) concentrations. A period of 24 h, a precursor concentration of 2 mM, and a pH of 8 were sufficient for high yield production of Ag-NPs. The Ag-NPs showed pronounced antibacterial and antifungal activities against the selected organisms. Furthermore, Ag-NPs showed high photocatalytic degradability of MB dye (98%) after 8 h co-incubation of MB-contaminated water under sunlight. Furthermore, treated water samples showed good germination of Vicia faba. Overall, results suggest that Ag-NPs have a strong potential for fast dye degradation and therefore, these Ag-NPs can be used in the future on large scale for the complete degradation of hazardous dyes from polluted water.

## Data Availability

Not applicable.

## Supplementary Materials

The following supporting information can be downloaded at: https://www.mdpi.com/article/10.3390/life12091331/s1, Table S1: Biochemical properties of *Cytobacillus firmus* strain MAE14 as indicated by the bioMérieux VITAK2 system.
