# Supplementary material for: Photocatalytic and Antimicrobial Activities of Biosynthesized Silver Nanoparticles Using Cytobacillus firmus
Source: Life (Basel). 2022 Aug 28;12(9):1331. doi: 10.3390/life12091331 (PMC9500943; doi:10.3390/life12091331)
Supplement: Supplementary file 1 [file life-12-01331-s001.zip › life-1875363-supplementary.pdf]

**Supplementary Table S1.** Biochemical properties of *Cytobacillus firmus* strain MAE14 as indicated by the bioMérieux VITAK2 system.

| Well | Test<br>G+ve endospore | abbreviation | Amount/Well<br>(μg) | Isolate code<br>MAE 14 |
|---|---|---|---|---|
| **1** | **β-xylosidase** | **BXYL** | 0.0324 | + |
| 3 | L-lysine-arylamidase | LysA | 0.0228 | - |
| 4 | L-aspartate arylamidase | AspA | 0.024 | + |
| 5 | leucine-arylamidase | LeuA | 0.0234 | + |
| 7 | phenylalanine arylamidase | PheA | 0.0264 | + |
| 8 | l-proline arylamidase | ProA | 0.0234 | - |
| 9 | β-galactosidase | BGAL | 0.036 | - |
| 10 | L-pyrrolydonyl-arylamidase | PyrA | 0.018 | + |
| 11 | α-galactosidase | AGAL | 0.036 | - |
| 12 | alanine arylamidase | AlaA | 0.0222 | + |
| 13 | tyrosine arylamidase | TyrA | 0.0282 | + |
| 14 | β-n-acetyl-glucosaminidase | BNAG | 0.0408 | - |
| 15 | ala-phe-pro arylamidase | APPA | 0.0384 | - |
| 18 | cyclodextrin | CDEX | 0.3 | - |
| 19 | d-galactose | dGAL | 0.3 | - |
| 21 | glycogen | GLYG | 0.1875 | - |
| 22 | myo-inositol | INO | 0.3 | - |
| 24 | methyl-a-d-glucopyranoside acidification | MdG | 0.3 | - |
| 25 | ellman | ELLM | 0.03 | + |
| 26 | methyl-d-xyloside | MdX | 0.3 | - |
| 27 | α-mannosidase | AMAN | 0.036 | - |
| 29 | maltotriose | MTE | 0.3 | - |
| 30 | glycine arylamidase | GlyA | 0.01 2 | + |
| 31 | d-mannitol | dMAN | 0.3 | - |
| 32 | d-mannose | dMNE | 0.3 | - |
| 34 | d-melezitose | dMLZ | 0.3 | - |
| 36 | n-acetyl-d-glucosamine | NAG | 0.3 | - |
| 37 | palatinose | PLE | 0.3 | - |
| 39 | L-rhamnose | IRHA | 0.3 | - |
| 41 | β-glucosidase | BGLU | 0.036 | - |
| 43 | β-mannosidase | BMAN | 0.036 | - |
| 44 | phosphoryl choline | PHC | 0.0366 | - |
| 45 | pyruvate | PVATE | 0.15 | + |
| 46 | α-glucosidase | AGLU | 0.036 | + |
| 47 | d-tagatose | dTAG | 0.3 | - |
| 48 | d-trehalose | dTRE | 0.3 | - |
| 50 | inulin | INU | 0.12 | - |
| 53 | d-glucose | dGLU | 0.3 | + |
| 54 | d-ribose | dRIB | 0.3 | + |
| 56 | putrescine assimilation | PSCNa | 0.201 | - |
| 58 | growth in 6.5% NaCl | NaCl 6.5% | 1.95 | + |
| 59 | kanamycin resistance | KAN | 0.006 | - |
| 60 | oleandomycin resistance | OLD | 0.003 | - |
| 61 | esculin hydrolysis | ESC | 0.0225 | - |
| 62 | tetrazolium red | TTZ | 0.0189 | + |

| | | | | |
|---|---|---|---|---|
| 63 | polymixinj3 resistance | POLYB_R | 0.00093 | - |
| | Probability (percent) | | | 94%<br>*Cytobacillus firmus* |
